# Coping with mortality salience: the role of connection thinking and afterlife beliefs in Chinese context

**DOI:** 10.3389/fpsyg.2023.1190906

**Published:** 2023-11-22

**Authors:** Kun Wang, Zhaoyang Sun, Yubo Hou, Mengchan Yuan

**Affiliations:** School of Psychological and Cognitive Sciences, Beijing Key Laboratory of Behavior and Mental Health, Peking University, Beijing, China

**Keywords:** terror management theory, mortality salience, death anxiety, afterlife beliefs, reincarnation beliefs, connection thinking

## Abstract

**Introduction:**

Grounded in Terror Management Theory (TMT), this research explored the influence of mortality salience on preferences for afterlife beliefs (reincarnation vs. resignation to fate) within a Chinese context. We also examined the mediating role of death anxiety and the moderating effects of connection thinking across different age groups.

**Methods:**

Across three experimental studies involving a cumulative sample of 485 Chinese participants, we primed individuals with thoughts of their own mortality and then assessed their death anxiety and proclivity toward reincarnation beliefs or resignation to fate. Connection thinking—a cognitive construct emphasizing relational interconnectedness—was also evaluated to ascertain its moderating impact.

**Results:**

The data revealed a pronounced preference for reincarnation beliefs as a distal defense mechanism following mortality salience, significantly mediated by death anxiety. The moderating role of connection thinking was also verified, but with age-related differences: among younger Chinese participants (age < 35), heightened connection thinking buffered against increased death anxiety triggered by mortality salience and thus mitigated its mediating role. Conversely, for older participants (age ≥ 35), amplified connection thinking exacerbated both the increased death anxiety and its mediating effect.

**Discussion:**

These findings contribute to TMT by elucidating the influence of death anxiety on the relationship between mortality salience and afterlife beliefs in the Chinese cultural context. They also enrich the literature on connection thinking by uncovering its moderating role. Moreover, our research yields practical implications for coping with mortality salience and alleviating existential anxiety, enhancing the understanding of these phenomena across different cultural and age groups.

## 1 Introduction

Death attitudes in China are closely tied to philosophical and religious traditions such as Confucianism, Buddhism, and Taoism. Buddhism views death as a transition and reincarnation as a way to cope with death-related anxiety (Deshpande et al., 2005; Tam et al., 2007). From a Confucian cultural perspective, it is believed that after death, one’s soul becomes a ghost that continues to exist in the world and coexists with the living, as this bestows meaning upon life (Yick and Gupta, 2002; Hsu et al., 2009; Chen, 2012). When facing death, Chinese people attach great importance to the dying process and funeral rites, hoping to arrive peacefully in the afterlife and enable their souls to continue to exist in a good state (Mjelde-Mossey and Chan, 2007; Liu and van Schalkwyk, 2019). The famous Chinese historian, Yu (2004) concluded that the Chinese belief in “soul to heaven and body to earth” was evident before the middle of the Western Han Dynasty (around the 1st century BC). The silk paintings found in the Mawangdui No.1 Han tomb, which depict scenes of heaven, earth, and hell, are a testament to Lady Dai’s endless imagination of the afterlife world, and show the representative cognition of the afterlife world and management of the fear of death among Chinese folk in the early Western Han Dynasty.

Studies have been conducted around Terror Management Theory (TMT) to investigate death-coping attitudes among atheists and theists, as well as individuals from different cultural backgrounds (Vail et al., 2010; Yen, 2013; Fan et al., 2022). Belief in an afterlife is an important death anxiety coping strategy for both theists and atheists. A study shows that even atheists’ beliefs in an afterlife can buffer their death anxiety (Heflick and Goldenberg, 2012). Other studies suggest that individuals in Eastern cultural backgrounds/Chinese people may not necessarily need a belief in an afterlife to buffer death anxiety. For example, Taiwanese participants showed tolerance and acceptance of death after being exposed to subliminal mortality salience (i.e., the participants’ awareness and cognitive recognition of their own death as an unavoidable event), as opposed to the solid cultural worldview defense seen in Western participants (Yen and Cheng, 2010). Asian individuals also tend to prefer using fatalistic and karma beliefs to cope with their awareness of death (Yen, 2013). This difference is likely due to Confucianism and Taoism teaching that life and death are determined by fate, such as the saying, “Life or death rests upon fate; wealth and rank lie in God’s hand.” Hence, death is seen as an inevitable result determined by one’s fate, leading Chinese individuals to reduce anxiety by rationalizing death (i.e., a proximal defense).

However, these systematic thoughts are the result of the rationalization of ancient philosophers, and may not fully represent the views of the general public. These conflicting findings from empirical studies and cultural beliefs highlight the need to examine the preferred coping mechanism for death anxiety among the Chinese after being exposed to mortality salience.

The Chinese culture also has an interconnected view of life and death, as seen in the famous quote by Confucius: “How can we know death without knowing life?” This connection is also reflected in ancient Chinese poems, such as the quatrain written by Qingzhao Li: “Be a man of men while you are alive, and soul of souls if you are dead.” In addition, ancestral worship has made childbirth and child-rearing a unique religious practice that provides Chinese people with a sense of meaning in life and a sense of continuation and heritage of family bloodlines to help them cope with their awareness of death (Qi, 2023). Another factor closely related to a sense of connection is intimate relationships, which have become an increasingly important buffer against death anxiety. Awareness of death prompts the establishment of intimate relationships because it enhances the sense of connection, enabling individuals to experience the greatest sense of connection and continuity in life (Plusnin et al., 2018). As a cognitive style, connection thinking has been measured and found to be mature and feasible (Choi et al., 2007; Hou et al., 2011, 2016), creating the conditions for this study to explore the role of connection thinking in managing awareness of death.

This study aims to investigate whether mortality salience enhances individuals’ reincarnation beliefs (afterlife beliefs) as a defense strategy among the Chinese, and whether this relationship is mediated by death anxiety. Additionally, we explore how the cognitive process of connection thinking moderates this mediation model.

### 1.1 Terror management theory

Terror Management Theory (TMT) was first proposed by psychologist Greenberg et al. (1986) and Solomon et al. (1991) based on the idea that human behavior is largely motivated by the avoidance of death, which was first suggested by anthropologist Becker (1973) in his book “*The Denial of Death.*” TMT suggests that when individuals are confronted with the reality of their own mortality, it elicits a fundamental psychological conflict, leading to death anxiety, the affective response of fear and dread arising from an awareness of mortality (Arrowood and Pope, 2014; Blomstrom et al., 2022).

Some indirect threat information related to death may trigger an individual’s awareness of death, e.g., retirement as an age-related transition may lead to more death anxiety and thus increased depressive symptoms (Segel-Karpas and Bergman, 2022). As research in the field of TMT has been enriched and expanded, it has also been found that exposure to the cues of a terrorist attack increases death anxiety and thus information avoidance, which decreases attitude polarization (Lee and Kim, 2021), suggesting that there is application and intervention value in death-related awareness at a wide range of levels, including political psychology. The COVID-19 pandemic, as an omnipresent mortality cue, is also a typical context for eliciting death anxiety (Hu et al., 2020; Shao et al., 2021). According to the analysis on the user-generated content of Twitter users, death anxiety peaked during the height of the pandemic (Barnes, 2021). Research in the field of neuroscience has identified brain regions and networks that are involved in processing emotional responses to death-related stimuli and threats to survival. For example, research has shown that the amygdala plays a critical role in processing fear and anxiety, including fear of death (Tanaka et al., 2022; Battaglia et al., 2023). Overall, successfully processing and coping with death anxiety is critical.

In recent years, TMT has been refined to include both negative and positive aspects of this psychological conflict, with some scholars suggesting that facing death can lead to personal growth (Taubman–Ben-Ari and Noy, 2010; Wei et al., 2015). The arousal of death awareness influences individual behavior and decision-making (Ho and Shiu, 1995; Routledge et al., 2010). Death anxiety can lead to negative behavioral outcomes and psychological distress unless alleviated (Shao et al., 2021), and failure to buffer against death anxiety often leads to negative consequences, such as individual consequences that lead to depression and a sense of loss of control (Abdel-Khalek, 1997; Zaleskiewicz et al., 2013; Grossman et al., 2018), and group consequences such as derogation of the out-group (Baldwin and Wesley, 1996). TMT posits two main defense mechanisms for coping with the fear of death: cultural worldviews and self-esteem, among which cultural worldviews provide meaning and purpose for individuals, and positively identifying with these worldviews can reduce negative thoughts and emotions related to death (Arndt et al., 1997a,b; Greenberg et al., 1986; Schmeichel and Martens, 2005; Hansen et al., 2010; Routledge et al., 2010; Du and Jonas, 2015; Hohman and Hogg, 2015). The cultural worldview is a belief that symbolic immortality or literal immortality can be achieved by conforming to cultural values (Scott et al., 2021). Specifically, the awareness of death drives people to confirm their contributions to a meaningful world to acquire symbolic immortality, including the adherence to social norms and values, such as honest behavior (Schindler et al., 2019), increasing political divide and hostile reactions during the COVID-19 pandemic (Kwon and Park, 2022), and the wish to have offspring to carry on the ancestral (Qi, 2023). Moreover, religious beliefs in afterlife which offer literal immortality can also play a role (Kwon and Park, 2022). In recent years, the third buffering factor of death anxiety, intimate relationships, has received increasing attention (Plusnin et al., 2018).

TMT also proposes a dual-path defense model that includes proximal and distal defenses. According to the model proposed by Pyszczynski et al. (1999), when death-related thoughts become salient in consciousness, individuals first engage in proximal defenses, such as suppressing or rationalizing these thoughts directly to avoid anxiety. This leads to a decline in Death Thought Accessibility (DTA), which is a measure of the ease with which death-related thoughts can be retrieved from memory (Hayes et al., 2010; Naidu et al., 2022). However, if the salience of mortality is subliminal or the buffering mechanism is threatened, DTA can increase immediately (Kosloff et al., 2019). Moreover, the emotional response to death thoughts is not immediate (Lambert et al., 2014), and distal emotional responses, such as fear, only emerge after a delay (Routledge et al., 2010). Suppressing death-related thoughts is a universal human instinct, and this proximal defense mechanism may be similar across cultures. For example, when measuring death-coping attitudes immediately after mortality salience, Chinese individuals tend to be more fatalistic, which could be interpreted as repression and rationalization of death (Pyszczynski et al., 1999; Arndt et al., 2007; Yen and Cheng, 2010).

In distal defenses, individuals may use symbolic or literal concepts designated by their cultural worldviews to cope with unconscious anxiety related to death (Dechesne et al., 2003). However, the expression of these defenses may differ between Western and non-Western cultures (Ma-Kellams and Blascovich, 2012; Yen, 2013; Park and Pyszczynski, 2016).

### 1.2 Distal and proximal defenses: reincarnation beliefs and resignation to fate

Afterlife beliefs are recognized as defenses in Terror Management Theory (TMT; Heflick et al., 2015; Lifshin et al., 2018) that can reduce both implicit and explicit death anxiety (Jackson et al., 2018). In addition, religious individuals tend to have lower levels of death anxiety and depression (Alvarado et al., 1995; Morris and McAdie, 2009) and their afterlife beliefs can even reduce their death avoidance attitude, as found in a study of Chinese religious believers (Fan et al., 2022). These afterlife beliefs serve as a defense mechanism that provides a sense of literal eternity, extending an individual’s life and existence beyond death and providing a powerful buffering effect (Dechesne et al., 2003). Even for those who are not religious, the belief in an afterlife can still serve as an effective death-buffering defense (Heflick and Goldenberg, 2012).

Yen and Cheng (2010) studied the defenses of Taiwanese individuals through beliefs in reincarnation (i.e., the existence of life after death) and resignation to fate (i.e., tolerance and acceptance of death) and found that participants preferred to resign to fate to buffer death anxiety and that mortality salience could evoke beliefs in fatalism and karma. The study found that this effect was stronger in an Eastern cultural context, but was not present in Western participants even when the Eastern cultural context was initiated (Yen and Cheng, 2010; Yen, 2013). However, this study did not clarify the specific stage in the terror management process where resignation tendencies act and it is unclear whether the results reflect the “rationalized” perception of death or the naive understanding of the general public. Furthermore, subliminal approaches to mortality salience may induce different emotional responses and death attitudes compared to traditional approaches (Pyszczynski et al., 1999; Lambert et al., 2014; Mahoney et al., 2014).

Our study predicts that reincarnation beliefs serve as death anxiety buffers in the context of Chinese culture. We consider reincarnation beliefs function as a distal buffer, reducing death anxiety only after a delayed distraction task (Greenberg et al., 1994; Dechesne et al., 2003; Vess et al., 2009; Testoni et al., 2019). On the other hand, resignation to fate is more of a proximal defense (Hayes et al., 2010; Yen and Cheng, 2010). Therefore, it is necessary to test this hypothesis through the supraliminal mortality salience approach.

Additionally, death anxiety serves as a mediator between mortality salience and death-coping attitudes. Individuals experiencing death anxiety may develop self-protective withdrawal motivations and behaviors (Grant and Wade-Benzoni, 2009), which may lead them to adopt external defense strategies. According to the classical assumptions of Terror Management Theory, individuals resort to distal defenses when death anxiety cannot be alleviated (Arndt et al., 1997a,b; Kosloff et al., 2019), such as belief in the afterlife (Aday, 1985; Heflick et al., 2015; Lifshin et al., 2018; Fan et al., 2022). Based on this, we propose the following hypotheses:

*H1*: Mortality salience can evoke reincarnation beliefs (vs. resignation to fate) in Chinese individuals.

*H2*: Death anxiety mediates the effect of mortality salience on reincarnation beliefs.

### 1.3 Cultural differences in TMT

TMT is widely accepted as a significant theoretical framework for understanding the human response to the awareness of mortality. However, the proposal of TMT cannot be completely divorced from the religious views in Western culture. Despite some cross-cultural studies based on Eastern cultural samples in recent years (Tam et al., 2007; Routledge et al., 2010), most of these studies have merely replicated the stabilizing effects found in previous TMT research. These studies often overlook the differences in cultural constructs, leading to inconsistent results when the effect of worldview defense is applied to non-Western cultures.

Studies of Eastern cultural backgrounds and samples have shown that the psychological response to death can vary among cultures. For instance, Yen and Cheng (2010) observed that Taiwanese individuals did not display the same solid cultural worldview defensive behaviors as Western participants after subliminal exposure to death. Rather than showing a higher level of affirmation and maintenance of their culture, the Taiwanese participants displayed tolerance and acceptance of death. According to a study, there was no significant difference in death anxiety levels between the control group and the experimental group after mortality salience was induced among participants in China. This can be explained by the cultural norm in China that discourages direct expression of high levels of anxiety or inadequate coping mechanisms (Ho and Shiu, 1995).

Moreover, several studies have also found unique effects of intimacy and attachment in Eastern cultures. For example, Chinese participants were found to be more fearful of the death of a loved one than of their own death (Hui and Fung, 2008). Children were also found to relieve the death anxiety of Chinese parents (Zhou et al., 2009). Based on China’s unique fertility culture, the value placed on the continuation of descendants is not only a distal defense related to, but independent of, cultural worldview, self-esteem and intimacy, but also a proximal defense (Qi, 2023). Plusnin et al. (2021) suggested that avoidant attachment was a “panacea” for coping with death anxiety in interdependent cultures because it relieved individuals’ fear of “collective mortality” in collectivist cultures.

The type of self-esteem used in different cultures to buffer death anxiety can also vary. The differences in cultural worldviews determine the type of self-esteem, as is widely accepted in the academic community (Greenberg et al., 1986). Compared to individualist cultures, Chinese participants in collectivist cultures tend to use collective self-esteem or relational self-esteem to buffer death anxiety (Du et al., 2013). When considering a broader scope, the tendency to associate objects with objects and subjects with objects in cognition and perception may play a unique role in buffering death anxiety.

By considering the cultural and cognitive differences between Eastern and Western cultures, this study sought to expand the scope of TMT and provide a more comprehensive understanding of human responses to mortality awareness.

### 1.4 The moderating role of connection thinking

The thinking style has been recognized as a crucial dimension of cultural differences (Ma-Kellams and Blascovich, 2012; de Oliveira and Nisbett, 2017). From a cognitive perspective, Chinese individuals tend to adopt a holistic thinking style as opposed to an analytical one, perceiving “life and death” and “weal and woe” as collective properties of things, rather than examining the relationship between them separately. The holistic thinking style not only significantly affects an individual’s cognition and emotion, but also reflects the impact of culture on individuals within a cultural group, highlighting the fundamental differences in cultural and philosophical views. Ma-Kellams and Blascovich (2012) were the first to discover cross-cultural differences in individuals’ tendencies to pursue life and respond to mortality salience through their investigation of holistic and analytical thinking styles, which initiate corresponding cognitive characteristics.

Connection thinking is a component of holistic thinking that measures an individual’s ability to connect different things and see all aspects of a problem (Choi et al., 2007; Hou et al., 2011, 2016). It is proposed that connection thinking moderates the relationship between mortality salience and death anxiety. Firstly, higher connection thinking leads to increased openness (Hou et al., 2016), and openness is related to death anxiety. Open individuals reduce their defensive need for self-esteem and cultural worldviews, thus accepting death as a novel experience and alleviating death anxiety (Boyd et al., 2017, 2020).

Additionally, connection thinking is also linked to the third buffer of death anxiety, apart from self-esteem and cultural values, intimacy. Intimacy is even considered as a preferred buffer of death anxiety by some researchers, as it enhances the sense of connection, enabling individuals to experience the greatest sense of connection and continuity in life (Plusnin et al., 2018, 2020). Thus, mortality salience can motivate individuals to establish and maintain intimacy, while the breakdown of intimacy can increase death thinking (Mikulincer et al., 2003). Research has shown that group identity can alleviate death anxiety through intimate relationships (Xiao et al., 2022). Mortality salience can also enhance the defense mechanisms of healthcare providers by increasing their relationship satisfaction (Chen et al., 2021), and marriage can also buffer death-related thoughts (Yaakobi, 2018). Some novel perspectives have also found that negative life events such as unemployment can lead to increased death-related cognitions, but intimate relationships can mitigate this effect (McCabe and Daly, 2018). When intimacy is damaged, individuals may exhibit a “compensatory effect” based on cultural worldviews, such as stricter condemnation and punishment of out-groups and individuals with different cultural beliefs (Chatard et al., 2010; Bassett and Connelly, 2011; Weise et al., 2012; Leippe et al., 2017). Evidence from neuroscience suggests that the prefrontal cortex and anterior cingulate cortex are involved in cognitive control and emotion regulation, which may be relevant to understanding how connection thinking moderates the effect of mortality salience on death anxiety (Kim et al., 2021; Di Gregorio and Battaglia, 2023; Tajti et al., 2023). Our research has shifted its focus from the connection between individuals and groups to the interdependence and connections between objects and individuals, as well as objects and objects. This feature of the relationship and interdependence between objects and subjects, as well as between objects, is consistent with the concept of intimacy. Some studies have even used the Intimacy Experience Scale and the Self-Construal Scale to measure collectivism and individualism, respectively (Wang and Mallinckrodt, 2006). Thus, the sense of connection underlying intimacy may play a crucial role in reducing death anxiety.

Based on this, the present study posits that connection thinking is a critical factor in perceiving death anxiety after mortality salience. Consequently, the following hypotheses are proposed:

*H3*: Connection thinking moderates the relationship between mortality salience and death anxiety. Individuals with high connection thinking exhibit lower death anxiety after mortality salience, whereas those with low connection thinking display higher death anxiety.

*H4*: Connection thinking moderates the mediating effect of death anxiety on the relationship between mortality salience and reincarnation beliefs. Specifically, for individuals with low connection thinking, mortality salience increases death anxiety, leading to higher beliefs in reincarnation. However, for individuals with high connection thinking, this mediating effect will not occur.

**Figure 1:**
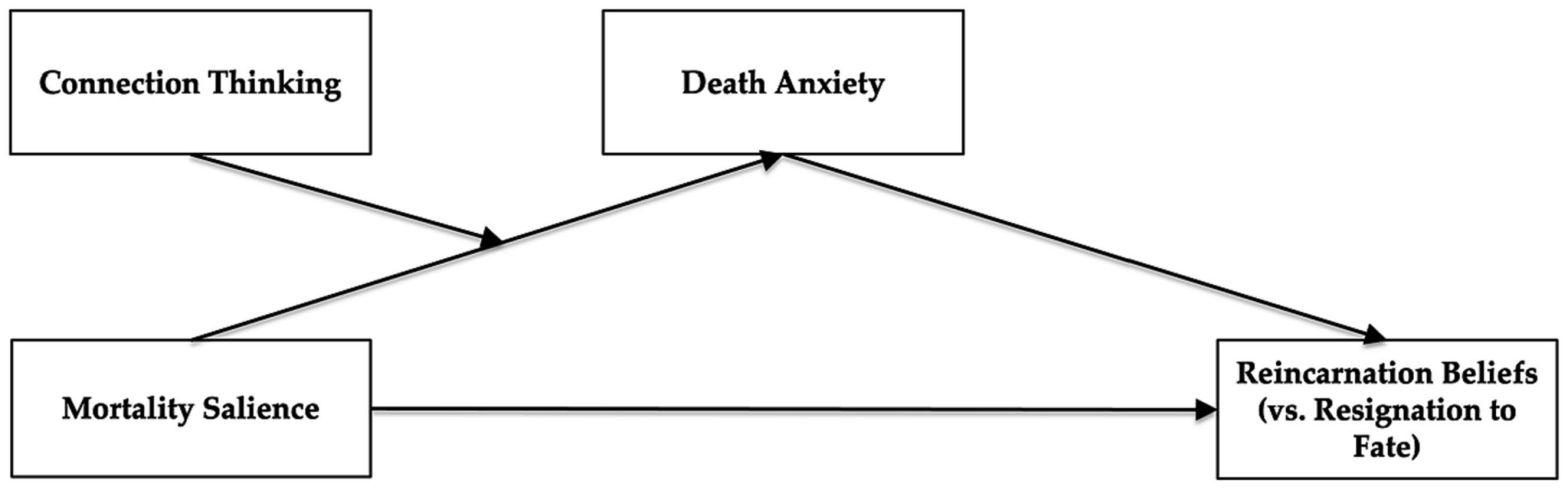
Conceptual model.

To test these hypotheses, two studies were conducted. Study 1 recruited participants online to examine the main effect of mortality salience on reincarnation beliefs and the mediating effect of death anxiety. Study 2 recruited participants from Peking University to replicate the results of Study 1, and provide initial evidence for the moderating effect of connection thinking. The proposed model is shown in Figure 1.

## 2 Study 1

Study 1 aimed to verify individuals’ tendency to pursue reincarnation beliefs as a distal defense strategy after mortality salience (H1) and the mediating role of death anxiety (H2).

### 2.1 Participants

Given that this study primarily employs regression analysis to verify the mediation model, the sample size was calculated using the Monte Carlo Power Analysis for Indirect Effects developed by Schoemann et al. (2017). Assuming correlations of 0.3 among the independent variable, dependent variable, and mediator, approximately 167 participants are required under α = 0.05 and power = 0.80 conditions. A total of 220 participants (74 males, *M*_age_ = 29.05, *SD*_age_ = 7.52) were recruited through the online platform Credamo. Participants who failed the attention checks or the text writing task, or had excessively short response time (<600 s), were excluded, leaving a sample of 173 participants (62 males, *M*_age_ = 29.83, *SD*_age_ = 7.97). These participants were divided into the MS (*N* = 90) or the control (*N* = 83) group. These participants were free from psychiatric diseases or any deadly diseases at the time of the examination.

### 2.2 Materials and procedures

The recruited participants were randomly grouped into the mortality salience group and the control group through the online system, after they confirmed they met the experimental requirements and provided informed consent. Upon completion of the experiment, participants were each remunerated 5 yuan RMB.

First, participants underwent a manipulation aimed at inducing mortality salience. This manipulation was conducted through a scrambled sentences task (Oyserman and Lee, 2008; Yan et al., 2021). All specific materials used in this study and the following studies were presented in the Supplementary material. In this task, participants in the MS group were presented with a sentence paragraph that was scrambled and described death, and were instructed to rank the scrambled sentence fragments. Conversely, the sentence paragraph for the control group described toothache. Subsequently, participants were asked an open-ended question: “When you think of your impending death/toothache, what physical and psychological feelings do you experience?.” Next, to ascertain the effectiveness of the manipulation, the intensity of death/fear/unpleasantness was then measured using an 11-point scale (0 = *not at all*, 10 = *very strong*). Following this, to further distract the participants, a number puzzle task and a number memory task were conducted. The number puzzle task was administered first, followed immediately by the number memory task, which required participants to remember a sequence of numbers. Completing both tasks took a minimum of 5 min in total.

Afterwards, we measured death anxiety by asking all participants to rate the degree of anxiety or uneasiness experienced on 7 items related to the death of self (α = 0.86; e.g., “The shortness of life”; 1 = *not anxious at all*, 6 = *very anxious*) based on their current genuine state and feelings. The assessment of death anxiety used the Your Own Death dimension of the Collett-Lester Fear of Death Scale version 3.0, revised by Lester and Abdel-Khalek (2003).

Moving on, we measured afterlife beliefs by asking participants to choose “Either/Or” items between reincarnation beliefs (“My death does not mean the end, even if the body dies, ‘life’ will continue to exist in some form”) and resignation to fate (“My death is the result of fate and I can only submit and accept it, so there is no so-called afterlife world”). Furthermore, we further assessed the degree of reincarnation beliefs using a 3-item and 6-point scale (α = 0.88; e.g., “believe there is life after death”; 1 = *strongly disagree*, 6 = *strongly agree*, Yen and Cheng, 2010). Finally, demographic variables including gender, age, education level, religious belief, and subjective socioeconomic status (Adler et al., 2000) were collected sequentially. Notably, the testing procedures were offered to all participants in the same order.

### 2.3 Results

#### 2.3.1 CMB and manipulation check

The results showed that the common method bias (CMB) was not present, as the first factor of Harman’s single-factor analysis only explained 38.77% of the variance, which was below the acceptable limit of 40% (Chang et al., 2020). The results of the independent-sample t-test showed that the manipulation of mortality salience was successful, as participants in the MS group (*M* = 7.78, *SD* = 1.53) perceived a higher degree of death compared to the control group (*M* = 3.47, *SD* = 2.52; *t*(1, 133.07) = 13.48, *p* < 0.001, Cohen’s *d* = 2.07, 95%CI [1.77, 2.37]). Participants in the MS group (*M* = 7.28, *SD* = 2.50) also perceived a higher degree of fear compared to the control group (*M* = 6.28, *SD* = 2.52; *t*(1, 171) = 2.62, *p* = 0.01, Cohen’s *d* = 0.40, 95%CI [0.10, 0.70]). However, there was no significant difference in perceived unpleasantness between the two groups [*t*(1, 171) = −1.25, *p* = 0.214]. There was no significant difference in levels of death anxiety between males (*M* = 4.08, *SD* = 1.02) and females (*M* = 4.06, *SD* = 1.10, *t*(1, 171) = −0.10, *p* = 0.92, Cohen’s *d* = −0.02, 95%CI [−0.33, 0.30]) in all samples, either for the control (*p* = 0.99) or MS group (*p* = 0.54).

#### 2.3.2 The effect of mortality salience on reincarnation beliefs

The results of the logistic regression analysis on “Either/Or” items showed that a greater proportion of participants in the MS group (66.7%) chose the option of reincarnation (0 = *resignation*, 1 = *reincarnation*) compared to the control group (50.6%), and this difference was statistically significant (*B* = 0.67, Exp (B) = 1.95, Wald = 4.56, *p* = 0.03). The results of the t-test on the continuous measure of reincarnation further showed that the participants in the MS group (*M* = 3.56, *SD* = 1.32) had a higher belief in reincarnation than those in the control group (*M* = 3.06, *SD* = 1.34; *t*(1, 171) = 2.49, *p* = 0.01, Cohen’s *d* = 0.38, 95%CI [0.08, 0.68]). Therefore, Hypothesis 1 was supported.

#### 2.3.3 Death anxiety serving as a mediator

**Figure 2:**
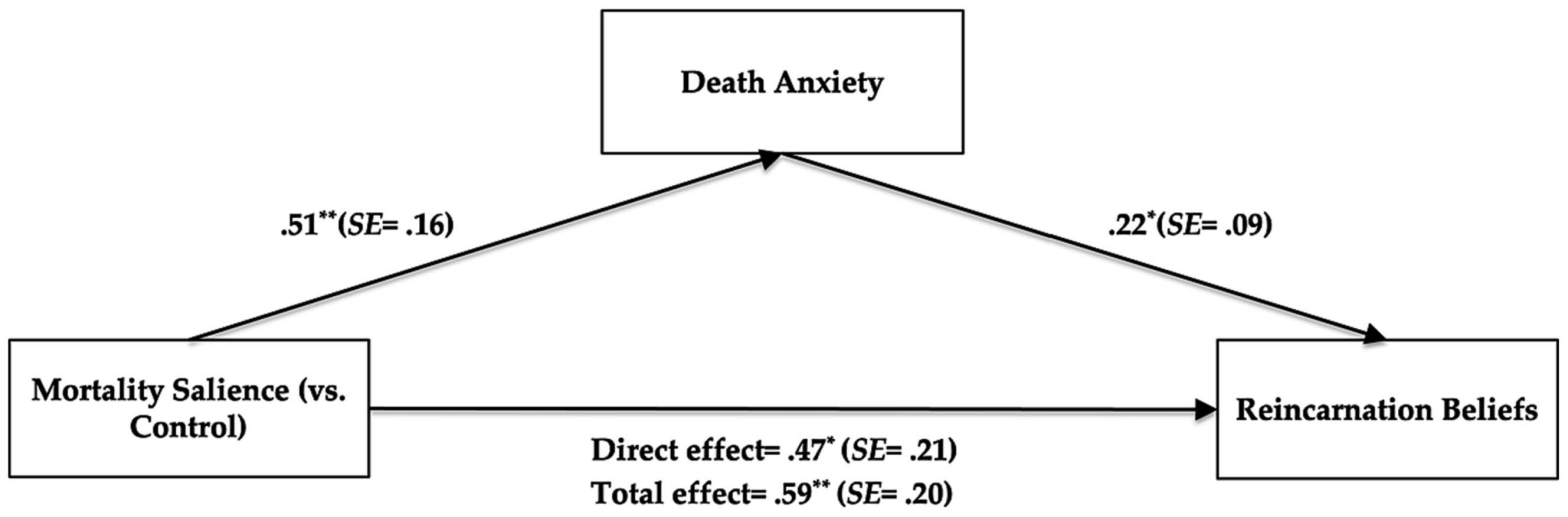
The results of mediation analysis. *p < 0.05, **p < 0.01.

We examined the mediating effect of death anxiety by conducting the mediation analysis in RStudio (Process model 4; 5,000 iterations; Hayes, 2018), in which the mortality salience was the independent variable (0 = *control*, 1 = *MS*), death anxiety was the mediator, reincarnation beliefs was the dependent variable, and demographics were controlling variables. The results showed that mortality salience had a significant positive effect on death anxiety (*B* = 0.51, *SE* = 0.16, *p* < 0.01, 95%CI [0.19, 0.83]), and death anxiety had a significant positive effect on reincarnation beliefs (*B* = 0.22, *SE* = 0.09, *p* = 0.02, 95%CI [0.04, 0.41]). The mediating effect of death anxiety was marginal significant (*B* = 0.11, *SE* = 0.07, *p* = 0.08, 95%CI [0.02, 0.29]; see Figure 2). These findings were verified even when demographic variables were controlled. Thus, Hypothesis 1 and Hypothesis 2 were supported.

## 3 Study 2

Study 2 aimed to replicate the results of Study 1 through another mortality salience materials. Moreover, this study, involving a sample of educated young adults (Age < 35), will examine the moderating role of connection thinking on the relationship between mortality salience and death anxiety (H3), as well as its impact on the mediating effect (H4).

### 3.1 Participants

Given the study’s focus on using multiple linear regression to test hypotheses, a sample size of 125 participants is determined using G*Power 3.1 (Faul et al., 2009). This sample size is needed to detect a manipulation test effect of small-to-medium magnitude (*f^2^* = 0.10) under α = 0.05 and power = 0.80 conditions. A total of 220 participants (82 males, *M*_age_ = 24.92, *SD*_age_ = 7.06) were recruited from Peking University. The 76 participants were excluded due to failed attention checks, not meeting text writing requirements, or short response time (<480 s). Four participants were excluded due to their age being greater than or equal to 35 years old. The remaining 140 participants (47 males, *M*_age_ = 22.66, *SD*_age_ = 2.85) were divided into the MS (*N* = 76) or the control (*N* = 64) group. These participants were free from psychiatric diseases or any deadly diseases at the time of the examination.

### 3.2 Materials and procedures

After recruiting participants and confirming that they met the experimental requirements and provided informed consent, the participants were randomly grouped. The following manipulations and measurements were then administered, and participants were paid 5 yuan RMB after completing the experiment.

We first manipulated mortality salience by asking participants to read a scenario description material developed by He et al. (2016) and write their feelings. Participants in the MS group read a description of cancer patients, while those in the control group read a description of patients with a toothache, both containing approximately 500 Chinese characters. Following their reading, participants answered two open questions: (1) What physical sensations do you experience when you consider your impending death (think about visiting the dentist)? (2) What psychological emotions do you feel when you contemplate your impending death (consider a visit to the dentist)? Then, the manipulation check was conducted, as in Study 1.

Following this, the delayed distraction task was carried out, as previously described in Study 1. After that, we assessed participants’ connection thinking using the relation subscale of the Chinese Holistic Thinking Style Scale (CHTTS, α = 0.56; 1 = *strongly disagree*, 7 = *strongly agree*; Hou et al., 2011, 2016). A higher score on this scale indicated a higher level of connection thinking. Then, death anxiety (α = 0.86), reincarnation beliefs (α = 0.93), and resignation to fate (α = 0.86) were successively assessed using 7-point scales (as in Study 1). Finally, demographic variables including gender, age, education level, religious beliefs, and subjective socioeconomic status (Adler et al., 2000) were collected.

### 3.3 Results

#### 3.3.1 CMB and manipulation check

We conducted an analysis examining the CMB of connection thinking, death anxiety, reincarnation beliefs, and orientation of resigning to fate, as measured by self-report questionnaires, through Harman’s single-factor analysis. The results showed that the first factor accounted for 28.60% of the variance, which suggested that there was no evidence of CMB in our study (Chang et al., 2020). Furthermore, the results of the independent-sample t-test indicated that participants in the MS group (*M* = 5.63, *SD* = 2.96) perceived a significantly higher level of death compared to those in the control group (*M* = 3.27, *SD* = 2.37; *t*(1, 137.66) = 5.26, *p* < 0.001, Cohen’s *d* = 0.88, 95%CI [0.55, 1.22]). A marginally significant difference emerged in perceived fear between the MS group (*M* = 6.17, *SD* = 3.01) and the control group (*M* = 5.19, *SD* = 3.13; *t*(1, 138) = 1.89, *p* = 0.06, Cohen’s *d* = 0.32, 95%CI [−0.01, 0.66]). There was no significant difference in perceived unpleasantness between the two groups [*t*(1, 138) = 1.29, *p* = 0.20]. Thus, our manipulation of mortality salience was successful. There was no significant difference in the level of death anxiety between males (*M* = 3.08, *SD* = 1.12) and females (*M* = 3.23, *SD* = 0.91, *t*(1, 138) = 0.82, *p* = 0.41, Cohen’s *d* = 0.15, 95%CI [−0.21, 0.50]) in all samples, either for the control (*p* = 0.24) or MS group (*p* = 0.99). There was no significant difference in the level of connection thinking between males (*M* = 5.54, *SD* = 0.76) and females (*M* = 5.60, *SD* = 0.74, *t*(1, 138) = 0.43, *p* = 0.67, Cohen’s *d* = 0.08, 95%CI [−0.28, 0.43]). In addition, there was no significant difference in the level of connection thinking between the control (*M* = 5.64, *SD* = 0.73) and MS groups (*M* = 5.53, *SD* = 0.76, *t*(1, 138) = −0.72, *p* = 0.38, Cohen’s *d* = −0.15, 95%CI [−0.48, 0.19]), suggesting that the mortality salience did not have an effect on it and that the measured connection thinking was a stable cognitive trait.

#### 3.3.2 The effect of mortality salience on reincarnation beliefs

The results of the t-test showed that participants in the MS group (*M* = 3.73, *SD* = 1.78) demonstrated a higher level of reincarnation beliefs compared to the control group (*M* = 3.10, *SD* = 1.71; *t*(1, 138) = 2.10, *p* = 0.04, Cohen’s *d* = 0.36, 95%CI [0.02, 0.69]). However, there was no significant difference observed in the resignation tendency between the MS group (*M* = 3.57, *SD* = 1.50) and the control group (*M* = 3.39, *SD* = 1.56; *t*(1, 138) = 0.68, *p* = 0.50, Cohen’s *d* = 0.11, 95%CI [−0.22, 0.45]). This supported Hypothesis 1.

#### 3.3.3 Death anxiety serving as a mediator

The results of the mediation analysis indicated the effect of death anxiety served as a mediator between mortality salience and reincarnation beliefs (*B* = 0.21, *SE* = 0.11, *p* = 0.06, 95%CI [0.04, 0.50]), which was marginal significant even after controlling for all demographic variables. However, death anxiety did not have a significant mediating effect between mortality salience and resignation to fate (*B* = 0.07, *SE* = 0.07, *p* = 0.30, 95%CI [−0.02, 0.26]). These results suggested that when individuals experience mortality salience, their heightened death anxiety is a driving factor for seeking reincarnation, in support of Hypothesis 2.

#### 3.3.4 Connection thinking serving as a moderator

We conducted a moderated mediation analysis using RStudio (Process model 7; 5,000 iterations; Hayes, 2018). In this analysis, mortality salience was the independent variable (0 = *control*, 1 = *MS*), death anxiety was the mediator, reincarnation beliefs was the dependent variable, connection thinking was the moderator, and demographics were controlling variables. The results showed a significant interaction effect of mortality salience and connection thinking (*B* = −0.50, *SE* = 0.22, *p* = 0.03, 95%CI = [−0.94, −0.06]), implying that connection thinking moderated the impact of mortality salience on death anxiety.

**Figure 3:**
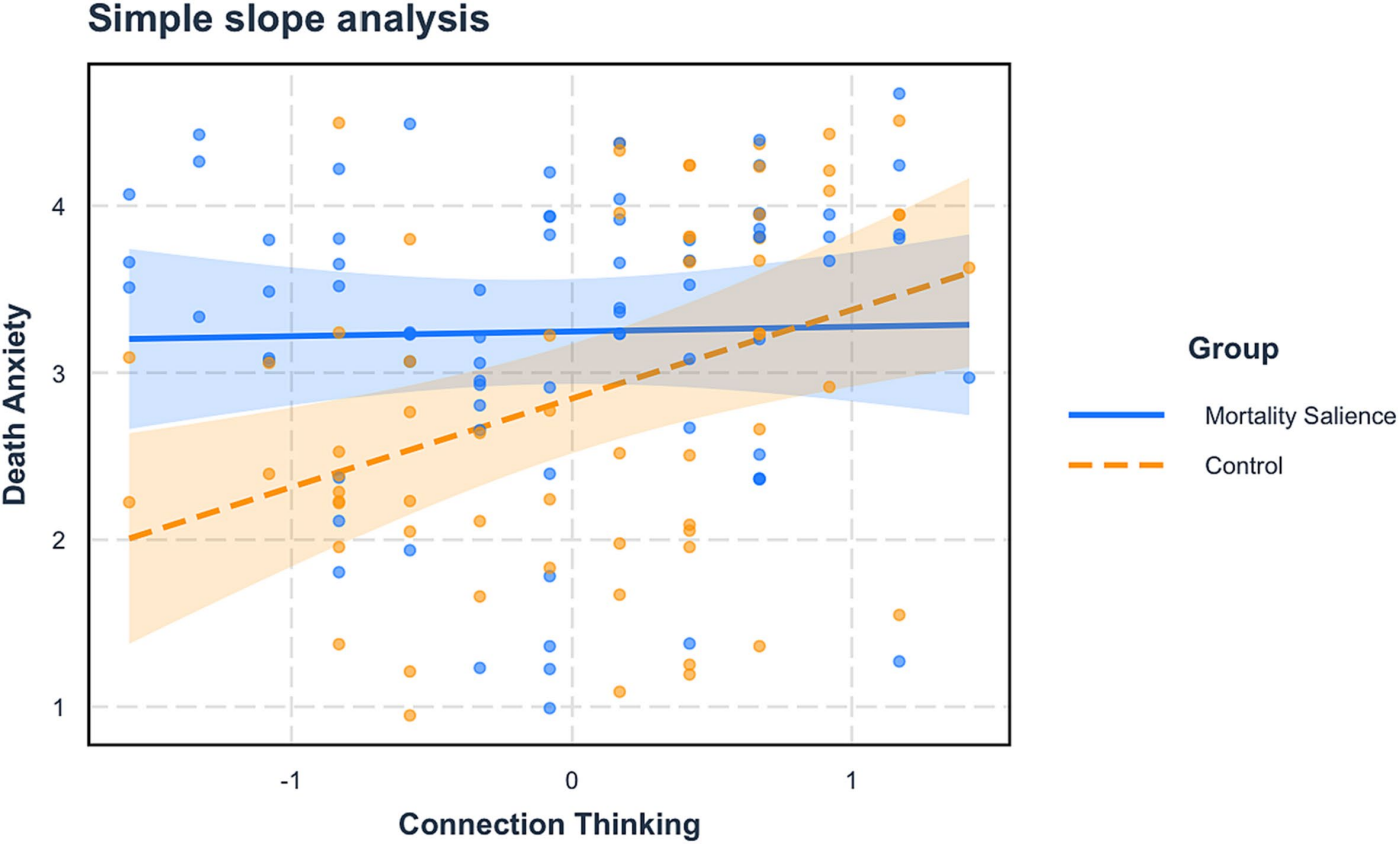
The moderating effect of connection thinking (Study 2).

A simple effect analysis was also performed, where connection thinking was ranked using cutoffs of the mean plus and minus one standard deviation. The results showed that when participants had a high level (*M + SD*) of connection thinking, mortality salience did not affect death anxiety (*B* = 0.03, *SE* = 0.23, *p* = 0.91, 95%CI [−0.44, 0.49]). However, when participants had a low level (*M - SD*) of connection thinking, mortality salience had a significant impact on death anxiety (*B* = 0.78, *SE* = 0.23, *p* = 0.001, 95%CI [0.32, 1.23], see Figure 3).

Additionally, connection thinking also moderated the mediating effect of death anxiety (*Moderated mediation index* = −0.27, *SE* = 0.14, 95%CI [−0.59, −0.04]). Specifically, when participants had a high level (*M + SD*) of connection thinking, the mediating effect was not significant (*B* = 0.02, *SE* = 0.13, *p* = 0.91, 95%CI [−0.23, 0.29]), while when participants had a low level (*M - SD*) of connection thinking, the mediating effect was significant (*B* = 0.41, *SE* = 0.17, *p* = 0.02, 95%CI [0.15, 0.84]). These overall effects remained significant even when controlling variables were excluded. Hence, Hypothesis 3 was supported.

## 4 Study 3

Given that the participant sample of Study 2 primarily consisted of well-educated young individuals, Study 3 aimed to replicate the findings of Study 2 among a more diverse population of middle-aged and older adults (Age ≥ 35), who often carry responsibilities such as marriage, eldercare, and child-rearing. Additionally, the mortality salience manipulation method employed in Study 1 was utilized to assess the robustness of outcomes arising from different mortality salience conditions.

### 4.1 Participants

Given the study’s focus on using multiple linear regression to test hypotheses, a sample size of 125 participants is determined using G*Power 3.1 (Faul et al., 2009). This sample size is needed to detect a manipulation test effect of small-to-medium magnitude (*f^2^* = 0.10) under α = 0.05 and power = 0.80 conditions. A total of 192 participants (84 males, *M*_age_ = 44.18, *SD*_age_ = 8.59) were recruited through the online platform Credamo. Participants who failed the attention checks or the text writing task, or had excessively short response time (<600 s), were excluded, leaving a sample of 172 participants (74 males, *M*_age_ = 45.10, *SD*_age_ = 7.90). These participants were divided into the MS (*N* = 85) or the control (*N* = 87) group. These participants were free from psychiatric diseases or any deadly diseases at the time of the examination.

### 4.2 Materials and procedures

After recruiting participants and confirming that they met the experimental requirements and provided informed consent, the participants were randomly grouped. The following manipulations and measurements were then administered, and participants were paid 5 yuan RMB after completing the experiment.

The current study replicated the design of Study 1 in terms of mortality salience, manipulation check, delayed distraction task, and sequence. Subsequent to the delayed distraction task, participants were assessed for death anxiety (α = 0.86), reincarnation beliefs (α = 0.91), resignation to fate (α = 0.89), and connection thinking (α = 0.49), utilizing the same measurement methods as in Study 2. Finally, demographic variables including gender, age, education level, religious beliefs, subjective socioeconomic status (Adler et al., 2000), caregiving responsibility for children, and caregiving responsibility for elderly relatives were collected.

### 4.3 Results

#### 4.3.1 CMB and manipulation check

We conducted an analysis examining the CMB of connection thinking, death anxiety, reincarnation beliefs, and orientation of resigning to fate, as measured by self-report questionnaires, through Harman’s single-factor analysis. The results showed that the first factor accounted for 26.27% of the variance, which suggested that there was no evidence of CMB in our study (Chang et al., 2020). Furthermore, the results of the independent-sample t-test indicated that participants in the MS group (*M* = 7.55, *SD* = 1.78) perceived a significantly higher level of death compared to those in the control group (*M* = 3.76, *SD* = 2.69; *t*(1, 149.31) = 10.93, *p* < 0.001, Cohen’s *d* = 1.66, 95%CI [1.36, 1.96]). A marginally significant difference emerged in perceived fear between the MS group (*M* = 6.93, *SD* = 2.40) and the control group (*M* = 6.24, *SD* = 2.82; *t*(1, 170) = 1.72, *p* = 0.09, Cohen’s *d* = 0.26, 95%CI [−0.04, 0.56]). There was no significant difference in unpleasantness (*t*(1, 170) = −1.0, *p* = 0.32) between the two groups. Thus, our manipulation of mortality salience was successful. There was no significant difference in the level of death anxiety between males (*M* = 3.92, *SD* = 1.22) and females (*M* = 3.87, *SD* = 1.11, *t*(1, 170) = −0.31, *p* = 0.76, Cohen’s *d* = −0.05, 95%CI [−0.35, 0.26]) in all samples, either for the control (*p* = 0.71) or MS group (*p* = 0.47). There was no significant difference in the level of connection thinking between males (*M* = 4.84, *SD* = 0.62) and females (*M* = 4.80, *SD* = 0.55, *t*(1, 170) = −0.46, *p* = 0.65, Cohen’s *d* = −0.07, 95%CI [−0.37, 0.23]). In addition, there was no significant difference in the level of connection thinking between the control (*M* = 4.80, *SD* = 0.57) and MS groups (*M* = 4.84, *SD* = 0.59, *t*(1, 170) = 0.44, *p* = 0.66, Cohen’s *d* = 0.07, 95%CI [−0.23, 0.37]), suggesting that the mortality salience did not have an effect on it and that the measured connection thinking was a stable cognitive trait.

#### 4.3.2 The effect of mortality salience on reincarnation beliefs

The t-test results indicated that participants in the MS group (*M* = 3.42, *SD* = 1.51) exhibited slightly higher reincarnation belief levels compared to the control group (*M* = 3.08, *SD* = 1.58), but this difference was not statistically significant (*t*(1, 170) = 1.44, *p* = 0.15, Cohen’s *d* = 0.22, 95%CI [−0.08, 0.52]). Additionally, there was no significant distinction in resignation tendency between the MS group (*M* = 3.72, *SD* = 1.35) and the control group (*M* = 3.67, *SD* = 1.46; *t*(1, 170) = 0.24, *p* = 0.81, Cohen’s *d* = 0.04, 95%CI [−0.26, 0.34]).

#### 4.3.3 Death anxiety serving as a mediator

The results of the mediation analysis indicated the effect of death anxiety served as a mediator between mortality salience and reincarnation beliefs (*B* = 0.21, *SE* = 0.09, *p* = 0.03, 95%CI [0.06, 0.43]), which was significant even after controlling for all demographic variables. However, death anxiety did not have a significant mediating effect between mortality salience and resignation to fate (*B* = 0.03, *SE* = 0.07, *p* = 0.71, 95%CI [−0.09, 0.18]). These results suggested that when individuals experience mortality salience, their heightened death anxiety is a driving factor for seeking reincarnation, in support of Hypothesis 2.

#### 4.3.4 Connection thinking serving as a moderator

We conducted a moderated mediation analysis using RStudio (Process model 7; 5,000 iterations; Hayes, 2018). In this analysis, mortality salience was the independent variable (0 = *control*, 1 = *MS*), death anxiety was the mediator, reincarnation beliefs was the dependent variable, connection thinking was the moderator, and demographics were controlling variables. The results showed a significant interaction effect of mortality salience and connection thinking (*B* = 0.86, *SE* = 0.29, *p* < 0.01, 95%CI = [0.28, 1.44]), implying that connection thinking moderated the impact of mortality salience on death anxiety.

**Figure 4:**
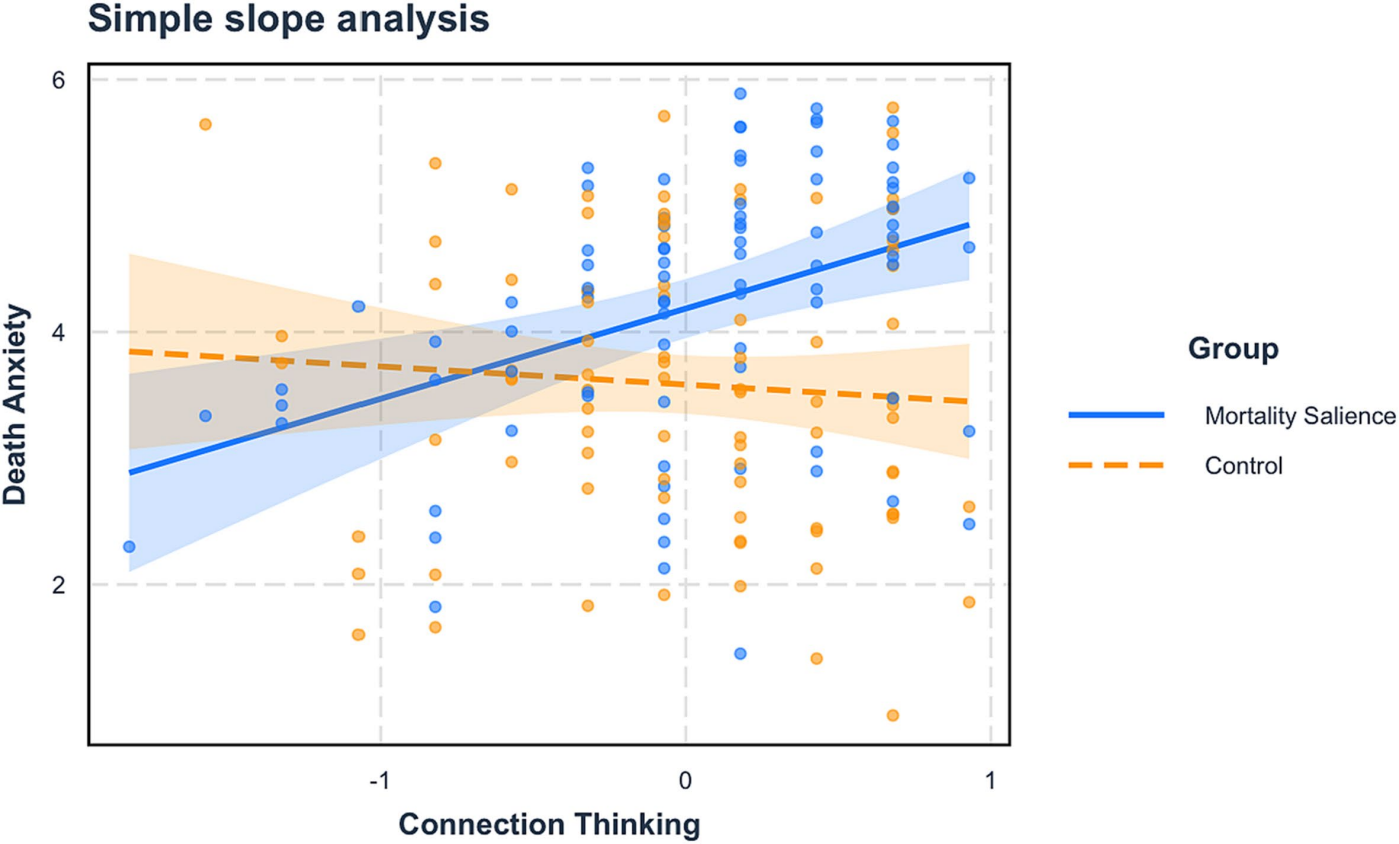
The moderating effect of connection thinking (Study 3).

A simple effect analysis was also performed, where connection thinking was ranked using cutoffs of the mean plus and minus one standard deviation. The results showed that when participants had a high level (*M + SD*) of connection thinking, mortality salience had a significant impact on death anxiety (*B* = 1.10, *SE* = 0.24, *p* < 0.001, 95%CI [0.63, 1.58]). However, when participants had a low level (*M - SD*) of connection thinking, mortality salience did not affect death anxiety (*B* = 0.10, *SE* = 0.24, *p* = 0.67, 95%CI [−0.37, 0.57], see Figure 4).

Additionally, connection thinking also moderated the mediating effect of death anxiety (*Moderated mediation index* = 0.29, *SE* = 0.13, 95%CI [0.06, 0.56]). Specifically, when participants had a high level (*M + SD*) of connection thinking, the mediating effect was significant (*B* = 0.33, *SE* = 0.14, *p* = 0.02, 95%CI [0.11, 0.65]), while when participants had a low level (*M – SD*) of connection thinking, the mediating effect was not significant (*B* = 0.03, *SE* = 0.07, *p* = 0.69, 95%CI [−0.09, 0.22]). Despite the presence of moderating effects of connection thinking on death anxiety and on the mediation effect, the direction is opposite to that of Study 2. This will be extensively discussed in the Discussion section.

## 5 General discussion

Numerous studies have explored the relationship between afterlife beliefs and death anxiety. The findings indicate that afterlife beliefs play a crucial role in alleviating death anxiety (Heflick et al., 2015; Jackson et al., 2018; Lifshin et al., 2018). However, other studies have yielded inconsistent results (Morris and McAdie, 2009; Yen and Cheng, 2010). The present study aimed to investigate the potential of reincarnation beliefs as a coping mechanism for death anxiety among Chinese individuals and the mediating role of death anxiety in the relationship between mortality salience and reincarnation beliefs. The study consisted of three parts. In Study 1, participants were asked to make a forced choice between reincarnation beliefs and resignation to fate after a delayed distraction task. The results showed that Chinese participants preferred reincarnation to resignation after experiencing mortality salience. Study 1 also measured reincarnation beliefs and tested the mediating model.

In Study 2, a sample of young and well-educated participants (aged under 35 years) was employed. Beyond successfully replicating the mediation mechanism of Study 1, a moderator—connection thinking—was introduced and measured following mortality salience. The results revealed that, when connection thinking was low, individuals in the mortality salience group exhibited significantly higher levels of death anxiety compared to the control group. However, when connection thinking was high, there were no significant differences in death anxiety levels between the two groups. This suggested that individuals with higher connection thinking exhibited reduced susceptibility to death anxiety after mortality salience, as opposed to those with lower connection thinking. Study 3 further examined the moderating role of connection thinking within the participant group aged 35 and above. Apart from confirming the mediation mechanism of Studies 1 and 2, an unexpected finding emerged: when connection thinking was low, there were no significant differences in death anxiety levels between the two groups; when connection thinking was high, the mortality salience group displayed significantly higher levels of death anxiety compared to the control group. This suggested that individuals with higher connection thinking exhibited heightened susceptibility to death anxiety after mortality salience, in contrast to those with lower connection thinking.

### 5.1 Theoretical implications

Our study makes several key contributions to the Terror Management Theory (TMT) literature. Firstly, we replicated and expanded upon previous findings on the role of mortality salience in the Chinese cultural context. Reincarnation beliefs significantly affected the distal defense stage of terror management in the mortality salience group. However, resignation to fate showed no significant difference between the groups. This contrasts with the results from Yen and Cheng (2010), who found that mortality salience enhanced resignation to fate in Taiwanese individuals. One possible explanation for this discrepancy is the close tie between the concept of reincarnation and death. This association might increase Death Thought Accessibility (DTA) and prevent Chinese individuals from using it as a proactive defense against death threats.

Additionally, the small sample sizes often used in TMT research may introduce sampling bias, and the effect size of the mortality salience effect is typically small in Eastern participants (Tam et al., 2007; Burke et al., 2010), which highlights the importance of our study that expanded the sample size for validation. Factors such as the era in which participants lived, including the COVID-19 pandemic’s heightened threat of death, must also be considered. Most notably, differences in research paradigms, such as our traditional supraliminal approach to mortality salience versus the subliminal approach of Yen and Cheng (2010), can lead to inconsistent results. Our method sequentially triggers both proximal and distal defenses, potentially yielding different outcomes (Arndt et al., 1997a,b; Pyszczynski et al., 1999; Kosloff et al., 2019). Overall, this research enhances cross-cultural comparisons by deepening insights into death threats and culture, and highlights the importance of nuanced differences in defense measurements and variable selection, which may lead to varied outcomes.

Previous research suggests that the Chinese tolerance and acceptance of death are rooted in Confucian and Taoist views of death as a predetermined outcome of personal fate and part of the life system. This perception of death displays a proximal defense strategy, as it avoids the threat of death through rationalization. The expression and rationalization of life and death struggles and traumas in Chinese literature also reflect this phenomenon (Tonglu, 2015). In East Asian countries like China, where secular beliefs are predominant and monotheistic religions are not the norm, the pursuit of afterlife beliefs is not an impossibility. In fact, mortality salience enhances the pursuit of beliefs in the afterlife and supernatural powers, regardless of religious or non-religious backgrounds. Research among Hong Kong Christians indicates that intrinsic religiosity aids Chinese individuals in alleviating death anxiety and enhancing their sense of life’s purpose (Hui and Fung, 2008). Among non-religious groups in China, beliefs in the afterlife significantly reduce individuals’ tendencies to avoid death (Fan et al., 2022). These findings affirm the efficacy of religious beliefs in mitigating existential threats. Religious beliefs offer individuals psychological security and the hope of literal immortality. Unlike secular beliefs, religious beliefs are all-encompassing and unfalsifiable, promising true and literal everlasting life. Mortality salience enhances beliefs in the afterlife, supernatural powers, human elevation from nature, and the distinction between mind and body. Consequently, religious beliefs concerning the afterlife are often considered the most fundamental and effective means of alleviating death anxiety (Vail et al., 2010, 2012). This perspective aligns with the views of young adults from different religions in the multicultural and multi-faith society of Peninsular Malaysia’s western region (Foong and Aziz, 2022). Moreover, this finding is consistent with the psychological effects of fear of death among Italian adolescents, where existential concerns indeed enhance the pursuit of religious spirituality and afterlife beliefs across different cultural backgrounds (Testoni et al., 2019).

Second, this study underscores the influence of cognitive traits in managing death threats. Soldatova and Zhukova (2018) and Coleman et al. (2019) both emphasized the role of secular beliefs, rooted in areas like science and education, as buffers against existential concerns. Whether through religious or secular ideologies, individuals derive personal meaning and bolster their self-esteem based on their cultural and personal beliefs. Our study’s findings align with this, suggesting that connection thinking, a secular thinking style deeply embedded in Chinese culture, might influence responses to mortality salience. Study 2 demonstrated that a high level of connection thinking style can mitigate death anxiety triggered by mortality salience, particularly within the young sample. This finding aligns with prior TMT studies, indicating that intimacy can alleviate death anxiety by fostering a sense of connection and continuity (Plusnin et al., 2018). Our study not only underscores the unique role of intimacy but also hints at deeper underlying coping mechanisms for death threats. Connection thinking, which involves drawing on external resources and integrating psychological resources, can help individuals manage death anxiety by satisfying the need for understanding and integration of cognition.

Interestingly, Study 2 found a small but significant positive correlation between connection thinking and death anxiety in the control group and Study 3 found a small but significant positive correlation between connection thinking and death anxiety in the mortality salience group, suggesting that high levels of connection thinking may amplify the impact of death threats. According to Plusnin et al. (2021), avoidant attachment can be a “panacea” for individuals in interdependent cultures, as it reduces the fear of collective death. However, it is important to consider whether this coping approach may also result in general death anxiety rather than just self-specific death anxiety when considering the cumulative effect of “collective mortality.” The findings from the Study 2 and Study 3 presented underscore the differential impact of connection thinking on death anxiety in response to mortality salience across different age groups. Specifically, for younger individuals (below 35 years), high levels of connection thinking seem to buffer against the effect of mortality salience on death anxiety. In contrast, for older individuals (above 35 years), high levels of connection thinking amplify the effect of mortality salience on death anxiety.

Research indicates that reactions to mortality salience differ between younger and older adults. Maxfield et al. (2007) noted that older adults, due to their proximity to the end of life, often encounter more direct reminders of death, such as peer deaths or health challenges. These experiences shape their existential perceptions. Developmentally, while younger individuals are often preoccupied with immediate concerns and future goals, older adults tend to seek deeper life meanings, making them more sensitive to mortality’s broader implications. This developmental trajectory suggests that coping strategies for death anxiety evolve over time. Younger individuals might lean toward avoidance or denial, whereas older adults, especially those with high executive function, show a cognitive resource-dependent shift toward a more accepting stance on death (Maxfield et al., 2014). Thus, in our study, the heightened death anxiety observed in younger individuals with low connection thinking might stem from a lack of avoidance mechanisms. In contrast, middle-aged and older adults, who generally have a more open attitude toward death, experience increased death anxiety under mortality salience as their connection thinking intensifies.

From a developmental perspective, the cognitive and emotional capacities of individuals evolve as they age. Younger individuals, being in the “Intimacy vs. Isolation” stage as posited by Erikson (1968), are primarily focused on forming intimate relationships and connections. This focus on connections might explain why younger individuals with a high level of connection thinking do not exhibit increased death anxiety in the face of mortality salience. Their cognitive style, which emphasizes connections, might serve as a buffer against existential threats, as they are more oriented toward forming bonds and relationships. On the other hand, older adults, navigating the “Generativity vs. Stagnation” stage, are more reflective and often seek deeper meaning and purpose in life. For them, a high level of connection thinking might amplify existential concerns. Their cognitive style, which has been shaped by years of experience and reflection, might make them more attuned to the broader implications of mortality, leading to heightened death anxiety when they engage in connection thinking (Salthouse, 1996). Furthermore, research has shown that age differences in cognitive styles and emotional responses can be attributed to reductions in working memory and processing speed (Salthouse, 1991). This suggests that older adults might process mortality salience differently compared to younger adults, leading to varied emotional responses. Additionally, older adults’ decision-making processes are influenced by both affective and deliberative information processes, which can further explain the observed age-related differences in the studies (Peters et al., 2007).

Terror Management Theory (TMT), as proposed by Greenberg et al. (1986), suggests that individuals employ various strategies to manage the existential anxiety arising from the awareness of mortality. One of these strategies is the bolstering of self-esteem and adherence to cultural worldviews, which provide a sense of meaning and continuity in the face of existential threats (Ayars et al., 2015). This suggests that individuals might derive personal meaning and bolster their self-esteem from different sources, depending on their cultural and personal beliefs. Chinese undergraduates, for instance, exhibit a unique mortality concept, emphasizing unfinished feelings, interpersonal relationships, and life reviews, reflecting Chinese cultural characteristics (Li and Liang, 2020). This cultural perspective might influence how connection thinking interacts with mortality salience. Furthermore, the emphasis on connection thinking in Chinese culture can be linked to traditional Chinese values, emphasizing harmony, relationships, and interconnectedness. In the context of the studies, connection thinking, deeply rooted in Chinese culture emphasizing harmony and interconnectedness, can be seen as a cultural worldview. This might influence how different age groups in China respond to existential threats, with younger individuals leveraging connection thinking as a protective mechanism, while older individuals might find it exacerbating their existential concerns.

This is further underscored by Fischer-Preßler et al. (2019), who observed that people use platforms like Twitter for collective sense-making and worldview validation in the face of existential threats. Moreover, cultural scripts, such as those related to time, can also shape reactions to mortality salience, as highlighted by McCabe and Bartholow (2019). Recent TMT studies in the Chinese context reveal that cultural influences significantly shape how individuals manage mortality anxiety. For instance, Chinese individuals employ both independent and interdependent self-esteem as anxiety buffers, reflecting a balance between individual and collective values (Du et al., 2013). This dual approach might elucidate the observed differences in our study, suggesting that Chinese individuals integrate both their cognitive styles and cultural values when confronting existential threats.

### 5.2 Practical implications

Our research also yields several practical implications. First, our findings highlight that death anxiety intensifies afterlife beliefs within the Chinese cultural context. Consequently, it is crucial for Chinese individuals facing severe death anxiety, such as those nearing the end of life, bereaved older spouses, and patients with advanced cancer, to receive support that aligns with their afterlife beliefs. Interpersonal relationships hold significant importance within Chinese culture. Thus, the loss of emotional connections with significant others can lead to particularly profound psychological stress for Chinese individuals. As such, religious beliefs can be especially suited to aiding Chinese individuals in coping with their own and their loved ones’ mortality, while also enhancing their sense of life purpose (Hui and Fung, 2008). This can alleviate their death anxiety, improving mental well-being. Effective interventions may include engaging in activities like reading books or watching films related to reincarnation, mindfulness meditation, cognitive-behavioral therapy tailored to address existential concerns, and psychotherapy sessions focusing on afterlife beliefs, and virtual reality simulations of out-of-body experiences (Blomstrom et al., 2022). Furthermore, it is crucial to offer assistance to individuals susceptible to death anxiety, including those with low self-esteem, individuals living alone, or those experiencing social isolation (Routledge et al., 2010). The global repercussions of the COVID-19 pandemic, which has intensified feelings of isolation, provide a unique backdrop to our study. Bouchoucha and Hutchinson (2022) explored the application of TMT during the pandemic, revealing that human connections can serve as a countermeasure against the pervasive feelings of isolation. This is especially pertinent for older Chinese adults, who, burdened by dual caregiving roles during such crises, might grapple with heightened death anxiety (Russell et al., 2020). The cultural emphasis on familial responsibilities in China further accentuates these feelings, with older individuals navigating the challenges of caregiving for medical conditions like stroke or cancer (Mor et al., 1994; Kalra et al., 2004). Kheibari (2019) further contextualized the relationship between loneliness, isolation, and suicidal attitudes within TMT, positing that intensified feelings of loneliness can amplify existential threats. This suggests that individuals, especially when isolated, become more susceptible to the effects of mortality salience (Meng et al., 2021). Our findings contribute to tailoring death-related education and support policies for diverse age groups, aiding socially isolated or relationally vulnerable populations in finding optimal beliefs and strategies to counter existential anxieties, such as engaging in religious or spiritual activities.

Secondly, our findings identified a moderating effect of connection thinking, particularly among younger individuals with lower levels of connection thinking, who are more susceptible to the impact of mortality salience. Consequently, interventions aimed at reducing exposure to death-related information can be promoted. For instance, they should be shielded from exposure to semantic or physical symbols of mortality, such as the direct mention of the word “death,” cemeteries, funerals, or any related text or videos that remind them of the inevitability of death. On the other hand, the discovery that individuals who exhibit strong connection thinking are less prone to experiencing death anxiety when confronted with mortality salience suggests that promoting connection thinking, involving practices such as exploring matters from diverse perspectives, embracing creative imagination, and actively engaging in extensive communication and learning to nurture an open-minded outlook, could serve as a viable strategy for mitigating death anxiety or dealing better with impending death. Contrastingly, for older middle-aged and elderly individuals, we can implement divergent practical suggestions and policies to alleviate their existential concerns, fostering life satisfaction and a sense of meaning.

### 5.3 Limitations and future research

This study has several limitations that should be acknowledged. Due to the COVID-19 pandemic, we conducted the study online, which led to a high dropout rate among participants (Study 1 = 21.36%, Study 2 = 36.36%, Study 3 = 10.42%). Future studies should consider conducting the experiment in a traditional offline laboratory setting to improve the robustness of the conclusions. Second, the internal consistency reliability of the connection thinking dimension scale in Study 2 (α = 0.56) and Study 3 (α = 0.49) was only considered acceptable, as discussed by Hou et al. (2016), who noted that scales for cultural constructs can have low reliability due to their abstract nature. However, this scale has good retest reliability, and its short length makes it easier to be used in research, compared to other similar tools. In the future, the Analysis-Holism Scale (AHS, Choi et al., 2007) or other local scales could be used to complement the results of cross-cultural comparisons. Additionally, the current study only focused on the moderating role of connection thinking in the Chinese cultural context, and it remains unclear whether these results can be applied in other Eastern or cultural backgrounds. Therefore, future research should focus on better understanding how individuals in different cultural backgrounds cope with death threats.

## 6 Conclusion

In the Chinese context, following supraliminal mortality salience, individuals tend to adopt reincarnation beliefs (i.e., faith in life after death) as a distal defense against death, rather than resignation. This effect is mediated by death anxiety. Moreover, connection thinking acts as a moderator, but this moderating effect exhibits variations across young and middle-aged to older adult populations. For younger Chinese individuals (age < 35), higher levels of connection thinking are associated with reduced susceptibility to death anxiety post-mortality salience. Connection thinking moderates this mediation; specifically, the mediation holds at low levels of connection thinking but not at high levels. Conversely, for older Chinese adults (age ≥ 35), elevated connection thinking predicts heightened death anxiety following mortality salience. Here, connection thinking also moderates the mediation, with the mediation being significant at high levels of connection thinking and non-significant at low levels.

## Data availability statement

The original contributions presented in the study are included in the article/Supplementary material, further inquiries can be directed to the corresponding author.

## Author contributions

KW: conceptualization, methodology, and formal analysis. KW and ZS: software, investigation, writing—original draft preparation, and visualization. KW and MY: validation and data curation. YH and KW: resources. KW, ZS, and MY: writing—review and editing. YH: supervision, project administration, and funding acquisition, YH. All authors have read and agreed to the published version of the manuscript.
