# Supplementary material for: Coping with mortality salience: the role of connection thinking and afterlife beliefs in Chinese context
Source: Front Psychol. 2023 Nov 22;14:1190906. doi: 10.3389/fpsyg.2023.1190906 (PMC10704099; doi:10.3389/fpsyg.2023.1190906)
Supplement: Supplementary file 1 [file Data_Sheet_1.docx]

**Coping with Mortality Salience: The Role of Connection Thinking and Afterlife Beliefs in Chinese Context**

**Supplementary Material**

[1. Study 1 3](#_Toc150702469)

[1.1 Manipulation of mortality salience 3](#_Toc150702470)

[1.2 Manipulation check 4](#_Toc150702471)

[1.3 Delayed distraction task 4](#_Toc150702472)

[1.4 Measurement of death anxiety 4](#_Toc150702473)

[1.5 Either-or question 5](#_Toc150702474)

[1.6 Measurement of reincarnation beliefs 5](#_Toc150702475)

[1.7 Measurement of subjective socioeconomic status 5](#_Toc150702476)

[1.8 The table showing the detailed results of mediation analysis 6](#_Toc150702477)

[2. Study 2 6](#_Toc150702478)

[2.1 Manipulation of mortality salience 6](#_Toc150702479)

[2.2 Manipulation check 8](#_Toc150702480)

[2.3 Delayed distraction task 8](#_Toc150702481)

[2.4 Measurement of connected thinking 8](#_Toc150702482)

[2.5 Measurement of death anxiety 8](#_Toc150702483)

[2.6 Measurement of reincarnation beliefs 8](#_Toc150702484)

[2.7 Measurement of resignation to fate 9](#_Toc150702485)

[2.8 Measurement of subjective socioeconomic status 9](#_Toc150702486)

[2.9 The table showing the detailed results of Study 2’s moderation analysis 9](#_Toc150702487)

[3. Study 3 9](#_Toc150702488)

[3.1 Manipulation of mortality salience 9](#_Toc150702489)

[3.2 Manipulation check 10](#_Toc150702490)

[3.3 Delayed distraction task 10](#_Toc150702491)

[3.4 Measurement of connected thinking 10](#_Toc150702492)

[3.5 Measurement of death anxiety 10](#_Toc150702493)

[3.6 Measurement of reincarnation beliefs 10](#_Toc150702494)

[3.7 Measurement of resignation to fate 10](#_Toc150702495)

[3.8 Measurement of subjective socioeconomic status 10](#_Toc150702496)

[3.9 The table showing the detailed results of Study 3’s moderation analysis 10](#_Toc150702497)

[4. Demographics information of all studies 11](#_Toc150702498)

# 1. Study 1

## 1.1 Manipulation of mortality salience

### *1.1.1 For the mortality salience group*

We are developing materials for future Chinese textbooks for primary and secondary schools. Here is a short article with the order of the sentences disordered. **Please connect each sentence fragment to form a complete and coherent sentence.** Please begin sorting them carefully.

The great French philosopher Pascal once said, “Man is but a reed, and a vapor, a drop of water suffices to kill him. However, man would still be nobler than his slayer; because he knows that he is dying, while the universe knows nothing of this.” Indeed, no one knows what will happen after he dies, but we have minds, and we can reason and speculate. Please imagine that one day you will rest in eternal sleep, you won’t be able to open your eyes or move your body, you won’t have time to do what you want to do, and you won’t have your career, family, friends, and love anymore. When you think of this, what emotions and physical feelings will you have?

We divide the above paragraph into 11 sentences in equal order. Each sentence is randomly divided into 3 fragments, and participants are asked to sort these sentence fragments.

**The open-ended question after the scrambled sentences task:**

When you think of your impending death, what physical and psychological feelings do you experience?

### *1.1.2 For the control group*

We are developing materials for future Chinese textbooks for primary and secondary schools. Here is a short article with the order of the sentences disordered. **Please connect each sentence fragment to form a complete and coherent sentence.** Please begin sorting them carefully.

Research in dental medicine has shown that neglecting oral hygiene can lead to various oral diseases, the most common of which is periodontitis and the resulting toothache. Subgingival scraping is a common and effective treatment for periodontitis, but this treatment often accompanies pain and discomfort. Dentists use the scraper head of the scraper to scrape away the deep-seated inflammatory material that accumulates below the gums. This can cause bleeding of the gums and is accompanied by severe pain, making patients restless. Therefore, during the subgingival scraping procedure, dentists inject anesthetics to relieve the pain. After the scaling is completed, patients still need to continue taking painkillers and antiphlogistic. Although subgingival scraping can cause bleeding and pain, it can effectively prevent further deterioration of the condition, such as tooth decay and loss. Have you gained a deeper understanding of oral hygiene? Imagine what kind of physical and psychological experience you would have when you have a toothache.

We divide the above paragraph into 11 sentences in equal order. Each sentence is randomly divided into 3 fragments, and participants are asked to sort these sentence fragments.

**The open-ended question after the scrambled sentences task:**

When you think of your toothache, what physical and psychological feelings do you experience?

## 1.2 Manipulation check

The intensity of death/fear/unpleasantness was measured using an 11-point Likert scale (0 = not at all, 10 = very strong).

## 1.3 Delayed distraction task

### *1.3.1 Number puzzle task*

**Please fill in numbers 1 to 6 in the circles below (each number can only appear once in each triangle)**, so that the sum of each edge in the first triangle is 9 and the sum of each edge in the second triangle is 10.

On the online platform, participants need to enter the numbers in the first and second triangles from top to bottom and left to right sequentially according to the instruction. The answer time is limited to 270 seconds (i.e., both the minimum stay time and the maximum stay time are set to 270 seconds), and the answer time will be displayed in real time. When the 270-second time limit is reached, the interface will automatically jump to the next page, regardless of whether the participant has completed the task.

### *1.3.2 Number memory task*

This question is designed to test your memory ability. Please remember this string of numbers within 30 seconds. After the countdown, **please click the next page immediately**. The string of numbers is **537-385-279-114-661**.

## 1.4 Measurement of death anxiety

Please rate your feelings about your own death in the following statements. Please answer as true to your feelings as possible after reading each item, without spending too much time thinking.

1. The total isolation of death
2. The shortness of life
3. Missing out on so much after you die
4. Dying young
5. How it will feel to be dead
6. Never thinking or experiencing anything again
7. The disintegration of your body after you die

Response categories are 1 = “not anxious at all” and 6 = “very anxious”.

## 1.5 Either-or question

Please choose which of the following descriptions better matches your view of “death”?

Option a: My death does not mean the end, even if the body dies, “life” will continue to exist in some form.

Option b: My death is the result of fate and I can only submit and accept it, so there is no so-called afterlife world.

## 1.6 Measurement of reincarnation beliefs

Please answer based on how you are feeling **right now**. **When facing my own death, I think:**

1. I believe there is life after death.
2. I believe the stories about reincarnation.
3. I believe in previous lives.

Response categories are 1 = “strongly disagree” and 6 = “strongly agree”.

## 1.7 Measurement of subjective socioeconomic status

The following image shows a 10-step ladder. Imagine that the ladder represents the different social classes that Chinese people belong to, with higher levels representing higher class status. For example, level 1 represents the lowest social class, where people have the worst living conditions, the lowest education level, the most undignified jobs, and the lowest income; level 10 represents the highest social class, where people have the best living conditions, the highest education level, the most respectable jobs, and the highest income. Please consider your own situation and
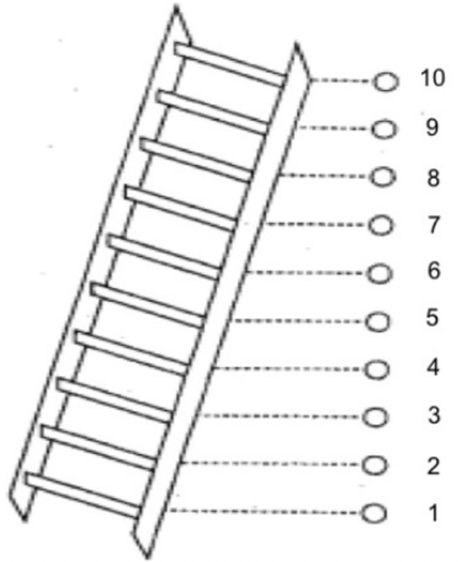
think about which step of the ladder you are located in.

## 1.8 The table showing the detailed results of mediation analysis

| Path | Effect | SE | p | 95%CI | | Conclusion |
| --- | --- | --- | --- | --- | --- | --- |
|  |  |  |  | BootLLCI | BootULCI |  |
| **Direct effect** |  |  |  |  |  |  |
| Mortality salience—Death anxiety 0 = control, 1 = mortality salience | 0.51 | 0.16 | < 0.01 | 0.19 | 0.83 | Significat |
| Death anxiety—Reincarnation beliefs | 0.22 | 0.09 | 0.02 | 0.04 | 0.41 | Significat |
| Mortality salience—Reincarnation beliefs | 0.47 | 0.21 | 0.02 | 0.07 | 0.87 | Significat |
| **Indirect effect** |  |  |  |  |  |  |
| Mortality salience—Death anxiety—Reincarnation beliefs | 0.11 | 0.07 | 0.08 | 0.02 | 0.29 | Marginal Significat |
| **Total effect** |  |  |  |  |  |  |
| Mortality salience—Reincarnation beliefs | 0.59 | 0.20 | < 0.01 | 0.21 | 0.97 | Significat |

# 2. Study 2

## 2.1 Manipulation of mortality salience

### *2.1.1 For the mortality salience group*

Psychologists have found that how a person faces the end of his life can reflect his personality. Therefore, please read this short article and then try to answer some related questions. Please read it word-for-word silently and deeply understand the situation described in the short article.

“So-and-so, it’s your turn!” I opened the door with a nervous heart and an old-looking attending doctor sat in front of the computer with the results of my CT scan on the screen. The doctor carefully examined the images and told me about my condition.

I remember it was two weeks ago, the weather was unusually cold that day. I forgot to wear an extra coat when I went out, and only realized I had caught a cold with a high fever after returning home. Hence I called the emergency room in the middle of the night to see the doctor and got the medicine, and thought it was just a common cold at that moment. However, a few days later, I still couldn’t stop coughing and felt quite uncomfortable, with a burning sensation in my chest, so I went to the hospital again for a thorough health checkup.

During the days before the report came out, while taking a shower, I accidentally found an abnormal lump in my abdomen that I had never noticed before, which was slightly smaller than the size of my hand. When I touched it, my heart sank, and my emotions became very complex. Although my intuition told me it was a tumor, I thought to myself, “I'm still so young, how could I have a tumor!” Therefore, I quickly rinsed my body, went back to my room, and kept going online to search for related information, desperately trying to find out the possibility that the lump was a tumor.

“It’s colon cancer, and your cancer cells have spread to your lungs.” said the doctor heavily. I walked out of the consulting room and cried bitterly on my parents’ shoulders. Soon, the nurse arranged for my hospitalization and the related treatment. A month passed, and I went through countless chemotherapy surgeries. I almost lost all of my hair, I lost my appetite, and had to rely on intravenous drips to supplement my strength. Whenever I thought of chemotherapy, I always felt cold and lonely. Gradually, I realized that people are actually very fragile, and perhaps death is always unexpected and unpredictable.

**The open questions to answer after reading the materials:**

1. What physical sensations do you experience when you consider your impending death?
2. What psychological emotions do you feel when you contemplate your impending death?

**Related questions:**

1. Please rate your level of understanding of the short article

1 = full understanding, 2 = mostly understanding, 3 = mostly not understanding, 4 = no understanding at all

1. What did the main character in the story go to the doctor for?

1 = fracture, 2 = asthma, 3 = cold, 4 = diarrhea

1. What kind of cancer did the main character in the story get?

1 = blood cancer, 2 = liver cancer, 3 = colorectal cancer, 4 = thyroid cancer

### *2.1.2 For the control group*

Psychologists have found that how a person faces physical pain can reflect his personality. Therefore, please read this short article and then try to answer some related questions. Please read it word-for-word silently and deeply understand the situation described in the short article.

“Open your mouth, it will be a little sore.” I lay on a chair next to a dentist wearing plastic gloves, a mask and a cap with only his eyes showing. He held a drill that kept squeaking and asked me to open my mouth to the limit, but all I could think about was how long it would last!

When I woke up a few days ago, I noticed that something was wrong with my teeth. When I drank ice water, I felt a soreness. Hence I took a mirror, opened my mouth to check my teeth, and found that the upper left molar had decayed and the lower wisdom tooth seemed to have grown crooked. Since I hadn’t been to the dentist for a long time, I searched online for recommended dentists, and soon I made an appointment by phone.

The waiting time for dental treatment always seemed long, and the pain in my teeth felt like countless tiny needles constantly pricking, and the tingling and soreness never stopped. I pressed my hand against my cheek tightly and kept diverting my attention to other places, but the pain pulled me back again. Finally, the nurse called my name, I had my X-ray taken and lay back on the chair. The nurse gave the X-ray to the dentist and told me to relax.

“Rinse your mouth!” The dentist said. I took a cup of water and rinsed my mouth, then spit it out, and the strong smell of blood permeated my mouth. Then the dentist told me that the plaque had decayed to the nerve and that I needed a nerve extraction, at which point my heart became even more torn. Soon, I watched the dentist inject the needle into my gums, and immediately an indescribable soreness emerged. After a while, my gums swelled up, and I tried to lick it with my tongue but found I couldn’t feel anything. Half an hour later, after the nerve was extracted, I walked back to the counter and made an appointment with the nurse for my next visit, but it all seemed like just the beginning of the pain.

**The open questions to answer after reading the materials:**

1. What physical sensations do you experience when you think about visiting the dentist?
2. What psychological emotions do you experience when considering a visit to the dentist?

**Related questions:**

1. Please rate your level of understanding of the short article

1 = full understanding, 2 = mostly understanding, 3 = mostly not understanding, 4 = no understanding at all

1. How did the main character in the story find the tooth decay?

1 = friend talked to him, 2 = found at dinner, 3 = drank ice water, 4 = health check

1. What operation did the doctor do for the main character?

1= denture replacement, 2 = teeth cleaning, 3 = nerve extraction, 4=tooth extraction

## 2.2 Manipulation check

Same as in Study 1.

## 2.3 Delayed distraction task

Same as in Study 1.

## 2.4 Measurement of connected thinking

What are your worldviews and views on life? For the following statements, please rate the extent to which you agree or disagree with them and select the corresponding number.

1. Many seemingly disconnected things are actually interrelated.
2. When an individual changes, they also influence those around them.
3. There often appears to be connections between things that appear unrelated.
4. I adapt myself to different situations and people to maintain positive relationships.

Response categories are 1 = “strongly disagree” and 7 = “strongly agree”.

## 2.5 Measurement of death anxiety

Same as in Study 1.

## 2.6 Measurement of reincarnation beliefs

Same as in Study 1.

## 2.7 Measurement of resignation to fate

Please answer based on how you are feeling right now. When facing my own death, I think:

1. Everything in life is due to destiny.
2. No matter how hard people try, what is going to happen will happen.
3. I agree with the old saying that life or death rest upon fate; wealth and rank lie in God’s hand.

Response categories are 1 = “strongly disagree” and 7 = “strongly agree”.

## 2.8 Measurement of subjective socioeconomic status

Same as in Study 1.

## 2.9 The table showing the detailed results of Study 2’s moderation analysis

| Path | Effect | SE | p | 95%CI | | Conclusion |
| --- | --- | --- | --- | --- | --- | --- |
|  |  |  |  | BootLLCI | BootULCI |  |
| **Main effect** |  |  |  |  |  |  |
| Mortality salience  0 = control, 1 = mortality salience | 0.45 | 0.29 | 0.13 | -0.13 | 1.03 | Not significant |
| Death anxiety | 0.53 | 0.15 | < 0.001 | 0.24 | 0.83 | Significant |
| Connection thinking | 0.06 | 0.20 | 0.76 | -0.34 | 0.46 | Not significant |
| **Moderating effect: on the relationship between mortality salience and death anxiety** | | | | | | |
| Mortality salience × Connection thinking | -0.50 | 0.22 | 0.03 | -0.94 | -0.06 | Significant |
| High connection thinking | 0.03 | 0.23 | 0.91 | -0.44 | 0.49 | Not significant |
| Low connection thinking | 0.78 | 0.23 | 0.001 | 0.32 | 1.23 | Significant |
| **Moderating effect: on the mediating effect of death anxiety** | | | | | | |
| Moderated mediation index | -0.27 | 0.14 |  | -0.59 | -0.04 | Significant |
| High connection thinking | 0.02 | 0.13 | 0.91 | -0.23 | 0.29 | Not significant |
| Low connection thinking | 0.41 | 0.17 | 0.02 | 0.15 | 0.84 | Significant |

# 3. Study 3

## 3.1 Manipulation of mortality salience

Same as in Study 1.

## 3.2 Manipulation check

Same as in Study 1.

## 3.3 Delayed distraction task

Same as in Study 1.

## 3.4 Measurement of connected thinking

Same as in Study 2.

## 3.5 Measurement of death anxiety

Same as in Study 1.

## 3.6 Measurement of reincarnation beliefs

Same as in Study 1.

## 3.7 Measurement of resignation to fate

Same as in Study 2.

## 3.8 Measurement of subjective socioeconomic status

Same as in Study 1.

## 3.9 The table showing the detailed results of Study 3’s moderation analysis

| Path | Effect | SE | p | 95%CI | | Conclusion |
| --- | --- | --- | --- | --- | --- | --- |
|  |  |  |  | BootLLCI | BootULCI |  |
| **Main effect** |  |  |  |  |  |  |
| Mortality salience  0 = control, 1 = mortality salience | 0.17 | 0.24 | 0.48 | -0.30 | 0.65 | Not significant |
| Death anxiety | 0.30 | 0.11 | < 0.01 | 0.09 | 0.51 | Significant |
| Connection thinking | 0.48 | 0.21 | 0.02 | 0.07 | 0.88 | Significant |
| **Moderating effect: on the relationship between mortality salience and death anxiety** | | | | | | |
| Mortality salience × Connection thinking | 0.86 | 0.29 | < 0.01 | 0.28 | 1.44 | Significant |
| High connection thinking | 1.10 | 0.24 | < 0.001 | 0.63 | 1.58 | Significant |
| Low connection thinking | 0.10 | 0.24 | 0.670 | -0.37 | 0.57 | Not Significant |
| **Moderating effect: on the mediating effect of death anxiety** | | | | | | |
| Moderated mediation index | 0.29 | 0.13 |  | 0.06 | 0.56 | Significant |
| High connection thinking | 0.33 | 0.14 | 0.02 | 0.11 | 0.65 | Significant |
| Low connection thinking | 0.03 | 0.07 | 0.69 | -0.09 | 0.22 | Not significant |

# 4. Demographics information of all studies

.

|  |  | Study 1 | | Study 2 | | Study 3 | |
| --- | --- | --- | --- | --- | --- | --- | --- |
|  |  | Number | Percentage | Number | Percentage | Number | Percentage |
| Gender | Male | 62 | 35.80% | 47 | 33.57% | 74 | 43.02% |
|  | Female | 111 | 64.20% | 93 | 66.43% | 98 | 56.98% |
| Age | 18 years-35 years | 147 | 85.00% | 140 | 100.00% | 0 | 0.00% |
|  | 36 years-60 years | 26 | 15.03% | 0 | 0.00% | 168 | 97.70% |
|  | > 60 years | 0 | 0.00% | 0 | 0.00% | 4 | 1.30% |
|  | Average age | 29.83 | / | 23.38 | / | 45.10 | / |
| Educational background | Junior high school and below | 2 | 1.20% | 0 | 0 | 5 | 2.91% |
|  | High school | 3 | 1.70% | 1 | 0.71% | 22 | 12.79% |
|  | Junior college/Undergraduate | 141 | 81.50% | 8 | 5.71% | 119 | 69.19% |
|  | Master | 26 | 15.00% | 92 | 65.71% | 24 | 13.95% |
|  | PhD | 1 | 0.60% | 39 | 27.86% | 2 | 1.16% |
| Religious belief | No | 163 | 94.22% | 123 | 87.86% | 128 | 74.42% |
|  | Yes | 10 | 5.78% | 17 | 12.14% | 44 | 25.58% |
